# Knowledge, attitudes, and support needs of obstetric and gynecological nurses and midwives toward perinatal mental health disorders screening in Central China: a multicenter cross-sectional survey

**DOI:** 10.3389/fpubh.2024.1424075

**Published:** 2024-10-09

**Authors:** Luyang Zhu, Xinlong Pan, Chunli Chen, Jianfei Chen, Yuanrui Pan, Xiaoli Chen, Zhijie Zou, Chengqiu Li

**Affiliations:** ^1^School of Nursing, Wuhan University, Wuhan, Hubei, China; ^2^Department of Obstetrics, Renmin Hospital, Wuhan University, Wuhan, Hubei, China; ^3^Department of Obstetrics and Gynecology, Renmin Hospital, Wuhan University, Wuhan, Hubei, China

**Keywords:** pregnant women, perinatal mental health disorders, knowledge, attitudes, support needs, medical staff

## Abstract

**Background:**

The perinatal period is a time of increased vulnerability regarding maternal mental health status. Although guidelines and policies have been published for perinatal mental health disorders (PMHDs) screening in China, the knowledge, attitudes, and support needs of nurses and midwives toward implementing mental health screening programs during pregnancy remain unclear. Thus, this study aimed to investigate the knowledge of PMHDs, attitudes and support needs related to implementing mental health screening during pregnancy among obstetrics and gynecology (OB/GYN) nurses and midwives in the central region of China while identifying the related influencing factors.

**Methods:**

A cross-sectional survey was conducted in 14 cities in Hubei, China, using convenience sampling from July to October 2023. The Chinese version of the Perinatal Mental Health Knowledge Questionnaire, the Chinese version of the Perinatal Mental Health Attitudes Scale, and the Health Care Facilities Support Needs Scale were used to investigate the PMHDs knowledge, attitudes, and support needs of OB/GYN nurses and midwives, respectively. Data were analyzed using SPSS version 27.0. Descriptive and inferential statistics were performed, with a *p*-value of <0.05 considered statistically significant.

**Results:**

The average scores for knowledge, attitudes, and support needs were 6.09 ± 1.99 (total score: 13), 47.67 ± 8.80 (total score: 80), and 29.35 ± 4.66 (total score: 35), respectively. After adjusting for years of nursing experience and years of obstetrics and gynecology nursing experience, the multivariate logistic regression analysis indicated that having mental health-related education or work experience [adjusted OR (aOR) = 1.43, *p* = 0.01], being midwives (aOR = 1.78, *p* < 0.001), and working in specialist maternity hospitals (aOR = 1.55, *p* < 0.001) were significantly associated with higher knowledge scores; having mental health related education or work experience (aOR = 1.59, *p* = 0.014) and working in specialist maternity hospitals (aOR = 1.42, *p* < 0.01) were significantly associated with higher support needs scores.

**Conclusion:**

OB/GYN nurses and midwives demonstrated insufficient knowledge and moderate attitudes toward PMHDs screening, and have great support need for PMHDs screening. To address these issues, medical organizations and relevant government sectors should enhance training for nurses and midwives on PMHDs and provide professional support to promote routine maternal mental health screening programs and improve perinatal mental health outcomes.

## Introduction

Maternal mental health is a key aspect of women’s overall well-being. The WHO defines maternal mental health as “a state of wellbeing where a mother can cope with life” stresses, work productively, and contribute to her community (1). Life-altering moments such as pregnancy, childbirth, and early parenthood can be stressful for women (2, 3). The perinatal period, encompassing pregnancy and the first year postpartum, particularly heightens maternal mental health vulnerability due to increased external attention, hormonal fluctuations, and changes in body image (4). Globally, almost 1 in 5 women will experience a mental health condition during pregnancy or in the year after birth (5–8). Recent meta-analyses found the prevalence of perinatal depression in mainland China to be 16.3%, with 19.7% for antenatal and 14.8% for postnatal depression (9). A survey in Wuhan, Hubei Province, found depression prevalence among pregnant women to be 30.77% at 3 months, 30.91% at 6 months, and 28.88% at 12 months postpartum (10), which was high.

Among women with perinatal mental health conditions, 20% will experience suicidal thoughts or engage in self-harm, about 20% of women may have suicidal thoughts or self-harm (11). Neglecting women’s mental health affects both their well-being and infants’ development (2). The adverse effects of poor perinatal mental health disorders (PMHDs) on the fetus may continue into childhood, increasing the risk of cognitive, motor, and emotional problems (12). Early identification and intervention of PMHDs can mitigate the risks to both mothers and babies (13).

The UK’s National Institute for Health and Clinical Excellence and the American Psychiatry Association recommend screening all women for anxiety and depression during pregnancy (14, 15). PMHDs screening has implemented in developed countries like the UK, US, and Australia (16–18). However, data showed that under 20% of women are assessed for mental health concerns during this period. Of less than 20% who screened positive for depression, only half received follow-up care, which reflects that healthcare providers may not be engaged in the important task of formally screening their pregnant and postpartum patients for mental health risks or providing referrals for mental health treatment (16).

Currently, there is no unified standard for who should conduct psychological screening for pregnant women. In Western public health systems, midwives play a key role in maternal mental health, leading to relevant studies (19). For example, Hauck et al. (20) and Noonan et al. (21) surveyed midwives’ knowledge, attitudes, and needs regarding PMHDs in Australia and Ireland, respectively. In China, obstetrics and gynecology (OB/GYN) nurses and midwives typically assume different but complementary roles in PMHDs management (22). Nurses need to assess the mental health of pregnant and postpartum women, provide support, educate about PMHDs, and assist in treatment while midwives focus primarily on women’s physiological health and childbirth management while monitor their emotional changes and guide them to seek professional mental health help when needed. Failing to understand their knowledge and needs may lead to delayed PMHD identification and treatment. The knowledge, attitudes, and practice model suggest that knowledge and attitudes influence behavior (23). In practice, knowledge and attitudes affect OB/GYN nurses’ and midwives’ involvement in perinatal mental health screening. Therefore, Understanding OB/GYN nurses’ and midwives’ knowledge, attitudes, and needs is crucial for effective intervention and screening programs.

In 2020, China’s National Health Commission recommended integrating depression screening into routine pregnancy and postnatal visits (24). However, implementation of these guidelines at the grassroots level remains limited. Currently, only Xiao et al. conducted a cross-sectional survey and qualitative interviews among obstetric medical staff in Shenzhen in 2018 (25) and 2019 (26), with 465 and 13 participants, respectively. Notably, Shenzhen is located in southern China and had a population of 17.63 million at the end of 2021 (27). Therefore, the findings are likely to be representative of obstetric medical staff in the region. Hubei Province, located in the central region of China, holds a core position in the strategy of central region development (28) with a population of 5,775,257 residents in 2022. In 2021, the annual birth population of Hubei Province was 404,000, with a birth rate of 6.98‰ (29). It is necessary to explore the current status of knowledge, attitudes, and support needs of OB/GYN medical staff in Hubei Province regarding PMHD screening projects.

Exploring influencing factors is crucial for understanding and improving healthcare professionals’ behavior. While some studies have examined the knowledge, attitudes, and support needs related to perinatal mental health screening, research on the influencing factors remains limited. Only Xiao et al.’s study (25) compared the scores of obstetric healthcare professionals with different characteristics regarding PMHDs, revealing that age, education level, years of experience, professional background, and mental health-related education or work experience significantly impacted their knowledge, attitudes, and support needs. Further investigation is needed into the influencing factors affecting the knowledge, attitudes, and support needs of OB/GYN nurses and midwives in PMHDs.

Thus, this study aimed to investigate the knowledge of PMHDs, attitudes, and support needs related to implementing mental health screening during pregnancy among OB/GYN nurses and midwives in the central region of China while identifying the related influencing factors.

## Materials and methods

### Study design, setting, and participants

This study was a cross-sectional survey. Using a convenient sampling method, nurses and midwives in OB/GYN from public hospitals in 14 cities in Hubei Province were invited to participate in the study from July to October 2023.

The inclusion criteria were as follows: (a) nurses and midwives employed in the OB/GYN ward or outpatient clinic of a public hospital in one of the 14 cities of Hubei Province; (b) had at least 1 year of experience working in the OB/GYN ward or outpatient clinic; and (c) were willing to participate in the study. The exclusion criteria were (a) nurses or midwives who were on leave (e.g., maternity leave, sick leave) or absent for other reasons (e.g., external training) during the survey period; (b) nurses or midwives who were undergoing rotation or advanced training in the OB/GYN ward or outpatient clinic during the survey period; and (c) nursing interns or students.

This study obtained ethical approval from the ethics board of the Ethics Committee of the Department of Medicine, Wuhan University (Whu-LFMD-IRB2023055).

### Sample size

Memon et al. (30) suggested that 15 to 20 observations per independent variable are strongly recommended in the multiple regression model. A total of 41 items were included in this study, which resulted in a sample size of 615 ~ 820. Considering a 20% attrition rate, 738 ~ 984 samples were needed. Additionally, Andrade (31) noted that the sample size may need to be larger in multicenter studies because of statistical noise due to variations in patient characteristics, nonspecific treatment characteristics, rating practices, environments, etc. between study centers. Therefore, our study ultimately included 1,271 participants.

### Data collection

An online self-administered questionnaire was used for data collection. The first page of the questionnaire provided a brief introduction on the background, objective, voluntary nature of participation, declarations of anonymity and confidentiality, and notes for completing questionnaire. Informed consent was obtained from each participant online by placing a question about their agreement to participate in the study at the beginning of the survey. After informed consent was obtained, the participant could continue to complete the questionnaire.

A pilot study was conducted with 30 nurses to verify the readability and wording of the questionnaire. Modifications were made on the basis of participants’ comments, which did not contradict the questionnaire’s design. The final questionnaire was then designed via the most extensive online survey platform in China, Wen Juan Xing (Changsha Ranxing Information Technology Co., Ltd., Hunan, China). Before the main study began, the online survey system was tested by the authors to check the performance of the online survey system in multiple browsers (including Chrome, internet Explorer, Microsoft edge, and Safari) and on different types of devices (including mobile phones, computers, and tablets).

The formal online questionnaire^1^ was distributed in the form of QR codes or web pages to WeChat (a popular social application in China) Workgroups between December 1, 2022, and February 20, 2023. To ensure the quality of the responses, duplicate questionnaires submitted by participants with the same IP address (*n* = 3) and respondents who took <100 s to complete the questionnaire (*n* = 26) were excluded. Finally, a total of 1,300 questionnaires were submitted, and 1,271 without missing values were included in the present work.

### Measurements

The online questionnaire designed for the study comprised four sections: demographic information, participants’ knowledge of PMHDs, participants’ attitudes toward PMHD, and participants’ support needs for PMHDs screening programs.

The demographic information questionnaire was self-designed by the researchers according to the research purpose and included age, years of working experience in OB/GYN, nature of the hospital (general hospitals or specialist maternity hospitals), hospital grade, title, category of personnel (nurse or midwife), and whether the respondent had mental health-related study or working experience (yes/no).

The Chinese version of the Perinatal Mental Health Knowledge Questionnaire was used to measure participants’ knowledge about PMHDs. The scale was first developed by Australian academics Hauck et al. (20) to measure midwives’ knowledge related to women’s perinatal psychology with 13 items. In 2017, Xiao et al. (25) translated the questionnaire into Chinese and conducted psychometric property tests. The Chinese version of the questionnaire is a reliable and valid instrument for assessing the PMHDs knowledge of medical staff, with a Cronbach’s *α* of 0.72. The final questionnaire consisted of 3 dimensions and 12 items. The six items related to the risks associated with mental illness included personal and family history, abuse and trauma, attachment, infant weight, hormonal protection and maternal age. Two items specifically addressed the Edinburgh Postnatal Depression Scale (EPDS). Four items related to the signs, symptoms and clinical management of mental illness were also included. Correct answers were scored as 1 point, and incorrect or unclear answers were scored as 0 points, for a total score of 12 points, with higher scores indicating greater knowledge of mental health during pregnancy. The overall evaluation was based on the percentage of correct answers, which was calculated as follows: total mean score/full score × 100%.

The Chinese version of the Perinatal Mental Health Attitudes Questionnaire was utilized to gauge participants’ attitudes toward PMHDs. Initially, developed by the Australian scholars McCall et al. (32). In 2002, this questionnaire was aimed at assessing general practitioners’ attitudes toward patients with depression and anxiety disorders. In 2012, Australian researchers Jones et al. (33) revised the questionnaire for use in measuring midwives’ attitudes toward psychological issues in pregnant women and new mothers. In 2017, Xiao et al. (25) translated and revised it into Chinese to evaluate healthcare professionals’ attitudes toward psychological health issues among pregnant women. The Chinese version of the questionnaire includes 15 items across two dimensions, namely, professional competence and comfort, and systemic issues. Examples of items include “My workload limits my ability to focus on depression or anxiety in pregnant women” and “The midwife/nurse/obstetrician should play the first role in dealing with anxiety and depression disorders in pregnant women.” A Likert scale ranging from “strongly disagree” to “strongly agree” was used for each item, with scores ranging from 1 to 5. The total score ranges from 15 to 75, with higher scores indicating a more positive attitude of healthcare professionals toward psychological issues among pregnant women.

The Health Care Facilities Support Needs Scale is designed to assess the support needs of healthcare professionals regarding the implementation of mental health screenings for pregnant women. The scale was developed by Xiao et al. in 2018 (25) on the basis of Bandura’s social cognitive theory with reference to the relevant literature and expert consultation. This scale consists of 7 items, all of which are related to the need for healthcare professionals to support the implementation of PMHDs screening, e.g., “I think there is a need for hospitals to have specialists in mental health.” Each item was rated on a 5-point Likert scale ranging from “strongly disagree” to “strongly agree,” with 1–5 points assigned. The total score ranges from 7 to 35, with a higher score indicating greater need from health care facilities. The Cronbach’s *α* of the scale was 0.93 (25).

### Statistical analysis

All the statistical analyses were done with SPSS 27.0. Categorical variables were presented as n (%), while continuous data following a normal distribution were described using means and standard deviations (SD). Data that deviated from a normal distribution were represented using median and interquartile range.

For normally distributed data, t-tests or ANOVA were used for intergroup comparisons, while non-normally distributed data were analyzed using the Mann–Whitney or Kruskal-Wallis H test. The mean knowledge, attitudes and support needs scores were used as the cut-off points. Variables with *p* values less than 0.05 in the univariate analyses were included in the multivariate logistic regression model. The results are reported as odds ratios (ORs) and 95% confidence intervals (CIs). A two-sided *p* value less than 0.05 was considered statistically significant.

## Results

### Demographic and baseline characteristics of the participants

In this study, a total of 1,271 questionnaires were collected, with 959 (75.06%) being nurses and 312 (24.55%) being midwives, 664 (52.24%) participants falling within the age bracket of 30–39 years, 1,129 (88.82%) attaining education up to the level of a bachelor’ s degree, and 371 (29.10%) working in specialist maternity hospitals. Of the total participants, only 300 (23.60%) had mental health-related education or work experience. Other information is detailed in Table 1.

**Table 1:** Demographic and baseline characteristics and differences in the knowledge, attitudes, and support needs of the participants (*n* = 1,271).

Variables	N (%)	Knowledge			Attitude			Support needs		
		Scores (Mean ± SD)	*t/F*	*P*	Scores (Mean ± SD)	*t/F*	*P*	Scores (Mean ± SD)	*t/F*	*P*
Age (years)										
18–29	364(28.64)	5.58 ± 1.84	2.021	0.133	47.22 ± 8.98	0.685	0.504	33.01 ± 5.60	3.426	0.053
30–39	664(52.24)	5.73 ± 1.88			47.88 ± 9.04			33.43 ± 5.44		
≥40	243(19.12)	5.89 ± 1.86			47.75 ± 7.83			33.37 ± 4.34		
Educational background										
College	142(11.17)	5.76 ± 1.88	0.58	0.63	47.07 ± 9.58	0.46	0.71	33.33 ± 5.48	0.53	0.66
Undergraduate	1,117(87.88)	5.71 ± 1.87			47.74 ± 8.70			33.46 ± 5.37		
Master	12(0.94)	5.50 ± 1.45			48.33 ± 9.26			33.67 ± 3.73		
Years of nursing experience										
1–5	212(16.68)	5.00(4.00,7.00)	10.41^a^	**0.02**	46.87 ± 9.30	0.89^b^	0.45	32.86 ± 5.78	3.15^b^	**0.02**
6–10	434(34.15)	6.00(4.00,7.00)			47.64 ± 9.15			33.13 ± 5.68		
11–20	449(35.33)	6.00(4.00,7.00)			48.07 ± 8.58			33.71 ± 5.03		
>20	176(13.85)	6.00(5.00,7.00)			47.68 ± 7.77			34.28 ± 4.78		
Years of obstetrics and gynecology nursing experience										
1–5	318(25.02)	5.00(4.00,7.00)	11.92^a^	**<0.01**	47.75 ± 9.34	0.50^b^	0.69	32.71 ± 5.69	4.50^b^	**<0.01**
6–10	436(34.30)	6.00(4.00,7.00)			47.34 ± 8.88			33.28 ± 5.65		
11–20	386(30.37)	6.00(4.00,7.00)			48.05 ± 9.01			33.96 ± 4.79		
>20	131(10.31)	6.00(5.00,7.00)			47.40 ± 6.19			34.32 ± 4.98		
Mental health related education or work experience										
No	971(76.40)	5.62 ± 1.88	−3.13	**<0.01**	47.81 ± 8.96	1.04	0.30	33.19 ± 5.46	−3.13	**<0.01**
Yes	300(23.60)	6.01 ± 1.80			47.21 ± 8.24			34.30 ± 4.97		
Mode of employment										
Labor dispatch	24(1.89)	5.79 ± 1.25	3.59	**0.03**	47.25 ± 5.38	0.05	0.96	32.25 ± 6.95	1.40	0.25
Contractual	1,036(81.51)	5.65 ± 1.90			47.70 ± 9.09			33.39 ± 5.43		
Official establishment	211(16.60)	6.02 ± 1.72			47.57 ± 7.60			33.90 ± 4.83		
Categories of personnel										
Nurse	959(75.06)	5.60 ± 1.87	8.68	**<0.001**	47.78 ± 9.18	2.06	0.13	33.29 ± 5.51	6.69	**<0.01**
Midwife	312(24.55)	6.08 ± 1.77			47.46 ± 7.38			34.04 ± 4.63		
Tittle										
Nurse	122(9.60)	5.67 ± 1.66	4.04	**<0.01**	47.61 ± 9.52	0.29	0.84	32.60 ± 5.76	4.19	**<0.01**
Nurse practitioner	489(38.47)	5.51 ± 1.98			47.47 ± 8.68			33.11 ± 5.41		
Nurse-in-charge	599(47.13)	5.84 ± 1.81			47.89 ± 8.98			33.73 ± 5.31		
Deputy director or chief nurse	61(4.80)	6.15 ± 1.64			47.16 ± 6.19			35.11 ± 4.15		
Nature of hospital										
General hospitals	900(70.81)	5.64 ± 1.86	2.33	**0.02**	47.59 ± 8.71	0.47	0.64	33.78 ± 5.43	−3.46	**<0.001**
Specialist maternity hospitals	371(29.1)	5.90 ± 1.87			47.85 ± 9.03			32.66 ± 5.12		

a *H-value.*

b *F-value.*

Bold values indicate *p* < 0.05, representing statistically significant results.

### Nurses’ and midwives’ knowledge of PMHDs

The average PMHDs knowledge score was 6.09 ± 1.99, with an average accuracy of 48.5%. The first dimension, related to risk factors, had the highest score. The dimensions of signs, symptoms and clinical management of mental illness had the lowest scores.

Specifically, the item “Women with postnatal depression often feel sad and cry (false)” had the lowest accuracy (3.38%). Additionally, the accuracy of the items “Postnatal depression will go away on its own but occasionally requires treatment (false)” and “Women must not breastfeed if taking medication for mental illness (false)” was also very low, at 9.76 and 17.62%, respectively (Table 2 and Supplementary Figure S1).

**Table 2:** Accuracy of PMHDs knowledge for nurses and midwives.

Items	Accuracy
Related risk factors	
Past personal history of mental illness is a risk for mental illness during childbearing(true)	93.31%
A history of abuse and trauma can increase the risk of mental illness in women(true)	91.90%
Women with mental illness may have attachment problems with their baby(true)	83.24%
Past family history of mental illness is not a risk for mental illness during childbearing(false)	57.12%
Hormones released during pregnancy protect against mental illness(false)	54.13%
Younger mothers are at greater risk of postnatal depression(false)	41.31%
Edinburgh Postnatal Depression Scale	
The EPDS is a useful screening tool for depression and anxiety(true)	49.05%
The cut off score for EPDS suggesting further assessment is 9(true)	43.27%
Signs, symptoms and clinical management of mental illness	
Women should not take psychiatric medication during pregnancy (false)	37.37%
Women must not breastfeed if taking medication for mental illness (false)	17.62%
Postnatal depression will go away on its own but occasionally requires treatment (false)	9.76%
Women with postnatal depression often feel sad and cry (false)	3.38%

### Nurses’ and midwives’ attitudes toward PMHDs

Nurses’ and midwives’ attitudes toward PMHDs ranged from 16 to 80 scores, with an average of 47.67 ± 8.80 scores. The item “I do not think I can tell the difference between a normal woman and a pregnant woman with psychological problems” had the lowest score (2.23 ± 0.96). The item “Pregnant women with symptoms of depression or anxiety should seek help from a psychologist or psychiatric specialist” had the highest score (4.10 ± 0.91). Additionally, the item “the midwife/nurse/obstetrician should play the first role in dealing with anxiety and depression disorders in pregnant women” also scored high (3.73 ± 0.98) (Table 3).

**Table 3:** Scores and responses of PMHDs attitudes for nurses and midwives.

Items, n(%)	Mean ± SD	Strongly agree	Agree	Neutral	Disagree	Strongly disagree
Professional competence and comfort						
My workload limits my ability to focus on depression or anxiety in pregnant women	2.72 ± 1.09	64(5.04)	254(19.98)	391(30.76)	384(30.21)	178(14.00)
I find psychological issues too complex and time-consuming to deal with	2.70 ± 1.13	60(4.72)	301(23.68)	297(23.37)	422(33.20)	191(15.03)
Pregnant women find it an invasion of privacy for medical staff to routinely ask them about their psychological condition	2.60 ± 1.08	50(3.93)	239(18.80)	322(25.33)	468(36.82)	192(15.11)
I work so tight a schedule that I cannot routinely assess the mental health of pregnant women	2.50 ± 1.06	47(3.70)	209(16.44)	278(21.87)	530(41.70)	207(16.29)
The hospital procedures (systems) I worked in prevented the medical staff from knowing enough about the psychological condition of pregnant women and giving adequate psychological care	2.40 ± 1.04	46(3.62)	158(12.43)	287(22.58)	547(43.34)	233(18.33)
I do not think I can tell the difference between a normal woman and a pregnant woman with psychological problems	2.23 ± 0.96	32(2.52)	120(9.44)	213(16.76)	651(51.22)	255(20.06)
Systemic issues						
Pregnant women with symptoms of depression or anxiety should seek help from a psychologist or psychiatric specialist	4.10 ± 0.91	446(35.09)	619(48.70)	135(10.62)	32(2.52)	39(3.07)
The midwife/nurse/obstetrician should play the first role in dealing with anxiety and depression disorders in pregnant women	3.73 ± 0.98	232(18.25)	657(51.69)	237(18.65)	92(7.24)	53(4.17)
I know very well when a pregnant woman has a psychological problem that needs to be referred to a medical professional	3.58 ± 0.97	212(16.68)	517(40.68)	372(29.27)	137(10.78)	33(2.60)
I think I’d be in a good position to counsel depressed pregnant women	3.34 ± 0.95	125(9.83)	441(34.70)	484(38.08)	177(13.93)	44(3.46)
I find it easier to deal with physical problems than mental ones	3.28 ± 1.06	103(8.10)	541(42.56)	314(24.70)	228(17.94)	85(6.69)
I’m comfortable with interventions for perinatal depression or anxiety	2.91 ± 0.95	71(5.59)	252(19.38)	498(39.18)	395(31.08)	55(4.33)
The priorities of the medical establishment now encourage me to focus on the problems that pregnant women present rather than identify the underlying problems	2.88 ± 1.15	89(7.00)	355(27.93)	298(23.45)	378(29.74)	151(11.88)
I feel uncomfortable asking pregnant women about mental disorders	2.58 ± 1.09	70(5.51)	206(16.21)	302(23.76)	505(39.73)	188(14.79)
I get frustrated when pregnant women with mental health problems consult me	2.49 ± 1.09	62(4.88)	188(14.79)	282(22.19)	523(41.15)	216(16.99)

### The support needs of nurses and midwives for the implementation of mental health screening for women during pregnancy

The support needs scores of the nurses and midwives ranged from 7 to 35, with an average of 29.35 ± 4.66 (Table 4). All the items had scores greater than 4 scores, among which the item “I think there is a need for hospitals to have specialists in mental health” had the highest score (4.26 ± 0.71).

**Table 4:** Scores and responses of PMHDs screening support needs for nurses.

Items, n(%)	Mean ± SD	Strongly agree	Agree	Neutral	Disagree	Strongly disagree
I think there is a need for hospitals to have specialists in mental health	4.26 ± 0.71	486(38.24)	664(52.24)	105(8.26)	2(0.16)	14(1.10)
I think maternal mental health should be assessed routinely	4.23 ± 0.71	455(35.80)	686(53.97)	108(8.50)	10(0.79)	12(0.94)
If the hospital offered training in mental health, I’d be happy to attend	4.22 ± 0.73	461(36.27)	663(52.16)	128(10.07)	5(0.39)	14(1.10)
Antenatal care providers should be equipped with skills to recognize maternal mental health	4.22 ± 0.72	445(35.01)	687(54.05)	121(9.52)	4(0.31)	14(1.10)
Health-care workers involved in antenatal care should be trained in the identification and management of maternal psychological problems	4.22 ± 0.70	441(34.70)	698(54.92)	117(9.21)	2(0.16)	13(1.02)
Antenatal care providers should be equipped with skills to treat maternal mental problems	4.13 ± 0.77	406(31.94)	669(52.64)	162(12.75)	19(1.49)	15(1.18)
Health-care workers should have access to information on dealing with perinatal maternal psychosocial problems	4.17 ± 0.73	413(32.49)	691(54.37)	144(11.33)	10(0.79)	13(1.02)

### Factors associated with knowledge, attitudes, and support needs

The results of the univariate analyses (Table 1) revealed that years of nursing experience, years of OB/GYN nursing experience, mental health-related education or work experience, mode of employment, category of personnel, title, and nature of hospital were the variables influencing the knowledge score (*p* < 0.05). Years of nursing experience, years of OB/GYN nursing experience, mental health-related education or work experience, category of personnel, title, and nature of the hospital were the influencing factors of support needs (*p* < 0.05). We found no significant associations between attitudes and any demographic or baseline characteristics (*p* > 0.05).

In the multivariate logistic regression analysis, variables with *p*-values less than 0.05 from the univariate analysis were included. Both the knowledge and support needs models were adjusted for years of nursing experience and years of obstetrics and gynecology nursing experience. The results revealed that having mental health related education or work experience (adjusted OR (aOR) = 1.43, 95% CI: [1.08 ~ 1.88], *p* = 0.01), being midwives (aOR = 1.78, 95% CI: [1.33 ~ 2.37], *p* < 0.001), and working in specialist maternity hospitals (aOR = 1.55, 95%CI: [1.20 ~ 2.02], *p* < 0.001) were significantly associated with higher knowledge scores (Table 5). Additionally, having mental health related education or work experience (aOR = 1.59, 95% CI: [1.09 ~ 2.31], *p* = 0.014) and working in specialist maternity hospitals (aOR = 1.42, 95%CI: [1.12 ~ 2.12], *p* < 0.01) were significantly associated with higher support needs scores (Table 6).

**Table 5:** Multivariate regression analysis (knowledge dimension).

Cut-off value: ≥6/<6	*Adjusted OR (95%CI)	*p*
Mental health related education or work experience		
No	Ref	
Yes	1.43(1.08 ~ 1.88)	**0.01**
Mode of employment		
Labor dispatch	Ref	
Contractual	1.14(0.49 ~ 2.61)	0.76
Official establishment	1.42(0.57 ~ 3.23)	0.45
Categories of personnel		
Nurse	Ref	
Midwife	1.78(1.33 ~ 2.37)	**<0.001**
Tittle		
Nurse	Ref	
Nurse practitioner	0.77(0.49 ~ 1.23)	0.28
Nurse-in-charge	0.91(0.54 ~ 1.54)	0.73
Deputy director or chief nurse	1.44(0.61 ~ 3.42)	0.41
Nature of hospital		
General hospitals	Ref	
Specialist maternity hospitals	1.55(1.20 ~ 2.02)	**<0.001**

*Adjusted for years of nursing experience, years of obstetrics and gynecology nursing experience.

Bold values indicate *p* < 0.05, representing statistically significant results.

**Table 6:** Multivariate regression analysis (support needs dimension),

Cut-off value: ≥29/<29	*Adjusted OR (95%CI)	*p*
Mental health related education or work experience		
No	Ref	
Yes	1.59(1.09 ~ 2.31)	**0.014**
Categories of personnel		
Nurse	Ref	
Midwife	1.28(0.88 ~ 1.84)	0.19
Tittle		
Nurse	Ref	
Nurse practitioner	1.03(0.59 ~ 1.81)	0.91
Nurse-in-charge	1.17(0.62 ~ 2.20)	0.64
Deputy director or chief nurse	1.56(0.76 ~ 2.14)	0.08
Nature of hospital		
General hospitals	Ref	
Specialist maternity hospitals	1.42(1.12 ~ 2.12)	**<0.01**

*Adjusted for years of nursing experience, years of obstetrics and gynecology nursing experience.

Bold values indicate *p* < 0.05, representing statistically significant results.

## Discussion

This is the first study to assess OB/GYN nurses’ and midwives’ knowledge, attitudes, and support needs regarding PMHDs in Central China. Results show that they had insufficient knowledge, moderate attitudes towards PMHDs screening, and great support needs for implementing mental health screening during pregnancy.

This study revealed that OB/GYN nurses’ and midwives’ knowledge about PMHDs was 6.09 ± 1.99, which was relatively low, close to that reported in Xiao et al.’s study (25) but lower than the mean score reported in Hauck’s survey of Australian midwives (8.27 ± 2.67) (20). This may be because perinatal mental health screening has been implemented in Australia for some time, whereas China is still in the early stages of this effort (24). Additionally, in our study, the item “Women with postnatal depression often feel sad and cry (false)” (only 3.38%) had the lowest accuracy, which is significantly lower than the accuracy rate reported in Australia (68.1%) (20). This discrepancy may be attributed to cultural differences. In China, pregnancy is traditionally viewed as a joyful event, leading to the suppression, underestimation, and unrecognition of negative emotions like sadness. Another contributing factor could be linked to a lack of relevant education, training, and work experience among nurses and midwives. Several studies indicate that both nurses and midwives often feel inadequately equipped in terms of their knowledge and skills concerning PMHDs due to insufficient training in this domain (34, 35). Limited access to continuous professional development opportunities can negatively impact individuals’ levels of knowledge related to PMHDs (35, 36). Therefore, there is a need to strengthen the training and education of nurses and midwives about PMHDs. Targeted training has been implemented in Australia (37), which showed that it can significantly enhance the knowledge, skills, and confidence of midwives. However, such training programs are not yet available in China. Thus, there is an urgent need for targeted educational interventions among Chinese healthcare providers.

In this study, nurses and midwives demonstrated moderate attitudes toward the mental health of perinatal women, recognizing that it is within their role to address anxiety and depression disorders in pregnant women. This aligns with Hauck’s study in Australia (20), where the majority of midwives (87.7%) agreed that assessing the mental health status of women in their care is part of their role. However, in contrast with Hauck’s study, in which less than half (42.7%) of participants believed that mental health issues during pregnancy should always be referred to a specialist, our study revealed that most participants felt that pregnant women experiencing symptoms of depression or anxiety should seek help from a psychologist or psychiatric specialist. Additionally, the average score for the item “I do not think I can tell the difference between a normal woman and a pregnant woman with psychological problems” was 2.23 ± 0.96, indicating that many participants lacked confidence in their ability to differentiate between normal and problematic psychological conditions. These findings suggest that while there is a willingness among OB/GYN nurses and midwives to engage in maternal mental health screening and care, there is also a significant gap in their professional knowledge, skills, and confidence. This is consistent with earlier results showing that they scored lowest in terms of knowledge about PMHDs signs, symptoms, and clinical management. Another possible explanation for this discrepancy could be the overall shortage of health resources in many Chinese hospitals, leading to overworked nurses and midwives who lack the time and energy to thoroughly assess the mental health status of pregnant women (26). Thus, it is essential to implement training programs focused on the signs, symptoms, and clinical management of PMHDs to enhance the knowledge and skills of OB/GYN nurses and midwives, thereby boosting their professional confidence. Studies in UK (38) and Ireland (39) have demonstrated that such training significantly improves the knowledge, skills, and confidence of midwives, including students. Additionally, improving human resource allocation is necessary to further support these healthcare professionals.

This study revealed that nurses and midwives expressed great support needs for PMHDs screening, aligning with findings from Xiao et al.’s studies (25, 26), which indicated that nurses and midwives require targeted training on PMHDs to enhance their competency in this area. Additionally, Xiao et al.’s study (26) highlighted other support needs among obstetric healthcare professionals, including the implementation of relevant policies to support perinatal mental health disorder screening, the establishment of multidisciplinary teams for mental health management, and the enhancement of public awareness of perinatal mental health disorders to reduce stigma and promote early detection and intervention. In our study, OB/GYN nurses and midwives also emphasized the necessity for hospitals to have specialists in mental health, likely due to the time-consuming nature of addressing psychological problems in pregnant women, given their busy schedules (26, 40). Moreover, considering their limited training in mental health, they prefer having psychologists in hospitals handle such issues, which aligns with the findings mentioned above. Given these findings, we recommend that China develop specific policies to address the identified support needs, such as implementing mandatory PMHDs training programs for OB/GYN nurses and midwives, increasing the integration of mental health specialists into obstetric care teams, and launching public awareness campaigns to facilitate the early identification and intervention of perinatal mental health issues. These steps are crucial for building a more supportive healthcare environment for both professionals and patients.

This study found that mental health-related education or work experience, personnel categories, and the nature of the hospital are important factors influencing the knowledge scores of OB/GYN nurses and midwives regarding PMHDs. Specifically, those with relevant education or work experience, midwives, and staff in specialist maternity hospitals demonstrated higher knowledge scores. This finding is consistent with Xiao’s study (25). Notably, our study revealed that midwives scored higher in knowledge than OB/GYN nurses, even though in traditional Chinese medical practice, ward nurses are generally more focused on the psychological care of pregnant women, while midwives primarily handle delivery-related care (22). This finding may suggest that midwives have increasingly emphasized perinatal mental health in their training and roles in recent years, or that they are more frequently exposed to related mental health issues in their work. Moreover, our study also showed that mental health-related education or work experience and the nature of the hospital were significantly associated with support needs scores. Specifically, nurses and midwives in specialist maternity hospitals had higher scores in both perinatal mental health knowledge and support needs compared to those in general hospitals. This may reflect the greater emphasis and higher standards placed on perinatal mental health training and evaluation in specialist hospitals. Our study further pointed out that those with mental health-related education or work experience not only had higher knowledge scores but also exhibited greater support needs. This result suggests that as knowledge levels increase, healthcare professionals become more aware of their limitations in perinatal mental health care, leading to a stronger desire for learning and support, thereby creating a positive feedback loop.

### Limitations

This study provides a snapshot of the prevailing knowledge, attitudes, and support needs among Chinese nurses and midwives. Thus, generalizability to other contexts may be limited. The data were collected solely in Hubei Province, China, and the sample may not be fully representative of the entire country, potentially introducing selection bias. Furthermore, the study relies on quantitative data without the supplementation of qualitative data beyond responses to open questions. Integrating qualitative insights could have offered a more comprehensive understanding of various perspectives. Importantly, relying solely on self-reported practices through survey methods may not fully capture the actual clinical practices and competency of midwives and nurses. Therefore, caution is warranted in interpreting the findings solely on the basis of this survey methodology. Finally, it is worth noting that there may be interactions between knowledge of, attitudes toward, and support needs for PMHDs. Unfortunately, this study did not identify a two-by-two correlation between these three factors. Therefore, further research is needed in the future.

## Conclusion

Currently, nurses and midwives specializing in OB/GYN in China exhibit insufficient knowledge about PMHD, with those having relevant education or work experience, being midwives, and staff in specialist maternity hospitals demonstrated higher knowledge scores. Furthermore, nurses and midwives display moderate attitudes toward PMHDs screening. There is a great need for support from government agencies regarding the implementation of PMHDs screening programs, with those having mental health-related education or work experience and in specialist maternity hospitals had higher scores.

Considering the potential role of nurses and midwives in PMHDs screening programs and the influencing factors mentioned above, it is essential to provide tailored training for nurses and midwives regarding PMHDs-related knowledge and skills. Moreover, providing professional support is crucial for facilitating the promotion of routine maternal mental health screening services in the future.

## Data Availability

The raw data supporting the conclusions of this article will be made available by the authors, without undue reservation.

## Footnotes

^1^ http://ww.wjx.cn

## References

[1] WHO. Promoting mental health: concepts, emerging evidence, practice. (2005). Available at: https://www.who.int/publications/i/item/9241562943 (Accessed March 15, 2024).

[2] Dowse E Chan S Ebert L Wynne O Thomas S Jones D. Impact of perinatal depression and anxiety on birth outcomes: a retrospective data analysis. Matern Child Health J. (2020) 24:718–26. doi: 10.1007/s10995-020-02906-6, PMID: 32303935

[3] Jahan N Went TR Sultan W Sapkota A Khurshid H Qureshi IA. Untreated depression during pregnancy and its effect on pregnancy outcomes: a systematic review. Cureus. (2021) 13:e17251. doi: 10.7759/cureus.17251, PMID: 34540477 PMC8448270

[4] Cheng C Chou Y Chang C Liou S. Trends of perinatal stress, anxiety, and depression and their prediction on postpartum depression. Int J Environ Res Public Health. (2021) 18:9307. doi: 10.3390/ijerph18179307, PMID: 34501906 PMC8431252

[5] Camoni L Gigantesco A Guzzini G Pellegrini E Mirabella F. Epidemiology of perinatal depression in Italy: systematic review and meta-analysis. Ann Dell'istituto Sup Sanita. (2023) 59:139–48. doi: 10.4415/ANN_23_02_07, PMID: 37337989

[6] Roddy Mitchell A Gordon H Atkinson J Lindquist A Walker SP Middleton A. Prevalence of perinatal anxiety and related disorders in low-and middle-income countries: a systematic review and meta-analysis. JAMA Netw Open. (2023) 6:e2343711. doi: 10.1001/jamanetworkopen.2023.43711, PMID: 37976063 PMC10656650

[7] Roddy Mitchell A Gordon H Lindquist A Walker SP Homer CSE Middleton A. Prevalence of perinatal depression in low-and middle-income countries: a systematic review and meta-analysis. JAMA Psychiatry. (2023) 80:425–31. doi: 10.1001/jamapsychiatry.2023.0069, PMID: 36884232 PMC9996459

[8] Yang L Sun J Nan Y Waqas A Nisar A Wang D. Prevalence of perinatal anxiety and its determinants in mainland China: a systematic review and meta-analysis. J Affect Disord. (2023) 323:193–203. doi: 10.1016/j.jad.2022.11.075, PMID: 36442655

[9] Nisar A Yin J Waqas A Bai X Wang D Rahman A. Prevalence of perinatal depression and its determinants in mainland China: a systematic review and meta-analysis. J Affect Disord. (2020) 277:1022–37. doi: 10.1016/j.jad.2020.07.046, PMID: 33065811

[10] Zhu Y Sun N Yin XX Fang PQ Gong YH. A follow-up study on the bi-directional relationship between postpartum depressive symptoms and parenting self-efficacy among women in China in the context of fertility policy adjustment: an empirical investigation based on Hubei Province. Popul Dev. (2023) 29:27–39.

[11] O'Connor EA Perdue LA Coppola EL Henninger ML Thomas RG Gaynes BN. Depression and suicide risk screening: updated evidence report and systematic review for the us preventive services task force. JAMA. (2023) 329:2068–85. doi: 10.1001/jama.2023.7787 37338873 PMC13533462

[12] Burger M Hoosain M Einspieler C Unger M Niehaus D. Maternal perinatal mental health and infant and toddler neurodevelopment - evidence from low and middle-income countries: a systematic review. J Affect Disord. (2020) 268:158–72. doi: 10.1016/j.jad.2020.03.023, PMID: 32174474

[13] Byatt N Yonkers KA. Addressing maternal mental health: progress, challenges, and potential solutions. Psychiatr News. (2021) 56. doi: 10.1176/appi.pn.2021.4.7

[14] National Institute for Health and Care Excellence (NICE). Antenatal and postnatal mental health: clinical management and service guidance. London, UK: National Collaborating Centre for Mental Health (2014). 31990493

[15] Opinion NAC. Screening for perinatal depression. Obstet Gynecol. (2018) 125:1268–71. doi: 10.1097/01.AOG.0000465192.34779.dc 25932866

[16] Diamond R. Perinatal mental health: 2022 year in review. (2022). Available at: https://www.psychologytoday.com/us/blog/preparing-parenthood/202212/perinatal-mental-health-2022-year-in-review (Accessed March 15, 2024).

[17] Moss KM Reilly N Dobson AJ Loxton D Tooth L Mishra GD. How rates of perinatal mental health screening in Australia have changed over time and which women are missing out. Aust N Z J Public Health. (2020) 44:301–6. doi: 10.1111/1753-6405.12999, PMID: 32510784

[18] Sidebottom A Vacquier M LaRusso E Erickson D Hardeman R. Perinatal depression screening practices in a large health system: identifying current state and assessing opportunities to provide more equitable care. Arch Womens Ment Health. (2021) 24:133–44. doi: 10.1007/s00737-020-01035-x, PMID: 32372299 PMC7929950

[19] Cibralic S Pickup W Diaz AM Kohlhoff J Karlov L Stylianakis A. The impact of midwifery continuity of care on maternal mental health: a narrative systematic review. Midwifery. (2023) 116:103546. doi: 10.1016/j.midw.2022.103546, PMID: 36375410

[20] Hauck YL Kelly G Dragovic M Butt J Whittaker P Badcock JC. Australian midwives’ knowledge, attitude and perceived learning needs around perinatal mental health. Midwifery. (2015) 31:247–55. doi: 10.1016/j.midw.2014.09.002, PMID: 25262025

[21] Noonan M Jomeen J Galvin R Doody O. Survey of midwives' perinatal mental health knowledge, confidence, attitudes and learning needs. Women Birth. (2018) 31:e358–66. doi: 10.1016/j.wombi.2018.02.002 29454664

[22] Li L Ding Y Mao LP. Progress of research on midwifery service models and human resource allocation at home and abroad. J Nurs. (2016) 31:15–7. doi: 10.3870/j.issn.1001-4152.2016.20.015

[23] Li Y Hu T Xia X Ge L. Knowledge, attitude, and practice toward photoaging in the Chinese population: a cross-sectional study. Sci Rep. (2024) 14:5196. doi: 10.1038/s41598-024-55691-5, PMID: 38431712 PMC10908786

[24] China Bureau of Disease Control and Prevention. Notice on exploring specialized services for the prevention and treatment of depression and Alzheimer's disease. General Office of the National Health and Health Commission (2020). Available at: http://www.nhc.gov.cn/jkj/s7914/202009/a63d8f82eb53451f97217bef0962b98f.shtml (Accessed March 15, 2024).

[25] Xiao X Lv CM Dl L Liu H Bai J Zhu SN. Analysis of the level of knowledge attitude and support needs of 465 obstetric healthcare professionals regarding mental health screening for women during pregnancy. J Nurs. (2018) 25:59–63. doi: 10.16460/j.issn1008-9969.2018.18.059

[26] Xiao X Ma H Zhu S Li Q Chen Y. The perceptions and attitudes of obstetric staff and midwives towards perinatal mental health disorders screening: a qualitative exploratory study in Shenzhen, China. BMC Nurs. (2023) 22:313. doi: 10.1186/s12912-023-01475-7, PMID: 37705010 PMC10498526

[27] National Bureau of Statistics Shenzhen Survey Team. Shenzhen national economic and social development statistics bulletin 2021. Shenzhen Bureau of Statistics. (2022). Available at: http://tjj.sz.gov.cn/gkmlpt/content/9/9763/post_9763042.html?eqid=cbeed34a000638a9000000036462f94d#4222 (Accessed March 12, 2024).

[28] Miao YN Yang DY. Analysis of S&T Resource Allocation Efficiency in central region based on comprehensive evaluation method. China Soft Sci. (2020) 3:134–49. doi: 10.3969/j.issn.1002-9753.2020.03.013

[29] National Bureau of Statistics Hubei General Investigation Team Hubei Province 2022 national economic and social development statistics bulletin Hubei Provincial Bureau of Statistics (2023). Available at: https://tjj.hubei.gov.cn/tjsj/tjgb/ndtjgb/qstjgb/202303/t20230316_4587528.shtml (Accessed March 15, 2024).

[30] Memon M Ting H Hwa C Ramayah T Chuah F Cham T. Sample size for survey research: review and recommendations. J Appl Struct Equ Model. (2020) 4:i–xx. doi: 10.47263/JASEM.4(2)01

[31] Andrade C. Sample size and its importance in research. Indian J Psychol Med. (2020) 42:102–3. doi: 10.4103/IJPSYM.IJPSYM_504_19, PMID: 31997873 PMC6970301

[32] McCall L Clarke DM Rowley G. A questionnaire to measure general practitioners' attitudes to their role in the management of patients with depression and anxiety. Aust Fam Physician. (2002) 31:299–303. PMID: 11926164

[33] Jones CJ Creedy DK Gamble JA. Australian midwives' awareness and management of antenatal and postpartum depression. Women Birth. (2012) 25:23–8. doi: 10.1016/j.wombi.2011.03.001, PMID: 21459691

[34] Arefadib N Cooklin A Nicholson J Shafiei T. Postnatal depression and anxiety screening and management by maternal and child health nurses in community settings: a scoping review. Midwifery. (2021) 100:103039. doi: 10.1016/j.midw.2021.103039, PMID: 34058681

[35] Johnson J Hope L Jones L Bradley E. A mixed methods study to understand perinatal mental healthcare referral decisions among midwives and health visitors in the UK. Front Psych. (2023) 14:1056987. doi: 10.3389/fpsyt.2023.1056987, PMID: 37377475 PMC10291319

[36] Silverwood V Nash A Chew-Graham CA Walsh-House J Sumathipala A Bartlam B. Healthcare professionals' perspectives on identifying and managing perinatal anxiety: a qualitative study. Br J Gen Pract. (2019) 69:e768–76. doi: 10.3399/bjgp19X706025, PMID: 31548296 PMC6758931

[37] McLachlan HL Forster DA Collins R Gunn J Hegarty K. Identifying and supporting women with psychosocial issues during the postnatal period: evaluating an educational intervention for midwives using a before-and-after survey. Midwifery. (2011) 27:723–30. doi: 10.1016/j.midw.2010.01.008, PMID: 20888094

[38] Davies L Page N Glover H Sudbury H. Developing a perinatal mental health module: an integrated care approach. The. Br J Midwifery. (2016) 24:118–21. doi: 10.12968/bjom.2016.24.2.118

[39] Higgins A Carroll M Sharek D. Impact of perinatal mental health education on student midwives' knowledge, skills and attitudes: a pre/post evaluation of a module of study. Nurse Educ Today. (2016) 36:364–9. doi: 10.1016/j.nedt.2015.09.007, PMID: 26431740

[40] Li YL Liu XM Meng YJ Li S. Survey and analysis of non-psychiatric healthcare workers' perception of mental health services in general hospitals. Nurs Res. (2018) 32:2968–71. doi: 10.12102/j.issn.1009-6493.2018.18.043

