# Supplementary figures and images for: Knowledge, attitudes, and support needs of obstetric and gynecological nurses and midwives toward perinatal mental health disorders screening in Central China: a multicenter cross-sectional survey
Source: Front Public Health. 2024 Oct 9;12:1424075. doi: 10.3389/fpubh.2024.1424075 (PMC11496250; doi:10.3389/fpubh.2024.1424075)

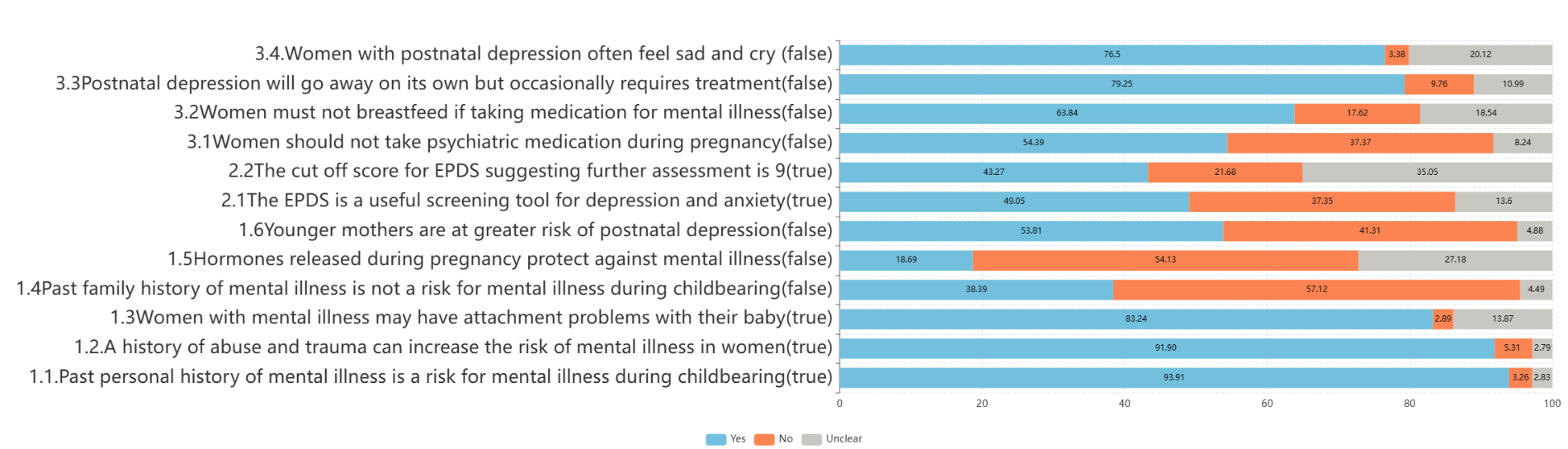


**Figure S1 Accuracy of PMHDs knowledge for nurses and midwives**

Supplement: Supplementary file 1 [file Data_Sheet_1.docx]
